# Sexually Transmitted Infection (STI) Incidence and Risk Factors Among People with HIV (PWH): Insights from a 13-Year Cohort Study in South Carolina

**DOI:** 10.1007/s10461-025-04744-5

**Published:** 2025-04-24

**Authors:** Salome-Joelle Gass, Shujie Chen, Jiajia Zhang, Bankole Olatosi

**Affiliations:** 1 Department of Health Services Policy & Management, University of South Carolina, Columbia, SC USA; 2 Department of Epidemiology and Biostatistics, University of South Carolina, Columbia, SC USA; 3 Arnold School of Public Health, South Carolina SmartState Center for Healthcare Quality, University of South Carolina, Columbia, SC USA

**Keywords:** HIV, Sexually transmitted infections, Incidence, Risk factors, South Carolina

## Abstract

The Ending the HIV Epidemic (EHE) initiative aims to reduce new HIV infections by 90% by 2030 in the United States (US). However, rising sexually transmitted infection (STI) rates exacerbate the bidirectional infection risk between HIV and STIs. Most research on STIs among people with HIV (PWH) has focused on high-risk groups, resulting in limited data on broader populations. This study addresses that gap by examining the incidence and risk factors for gonorrhea, chlamydia, and syphilis in a statewide cohort of PWH in South Carolina. Data from South Carolina’s HIV and STI surveillance systems were linked, and all PWH aged 18 and older who were diagnosed with HIV between 2007 to April 2018 were included. Cohort demographics were analyzed using descriptive statistics and chi-squared tests, and a Cox Proportional Hazards model examined time to first STI after HIV diagnosis. The study found an increase in the incidence of gonorrhea, chlamydia, and syphilis over the study period. Young adults, men, Black individuals, MSM, and urban residents were found to be at increased risk for STI diagnosis. Clinical risk factors associated with increased STI risk included lower initial CD4 counts, and higher initial viral loads. The findings underscore a considerable STI burden among PWH in South Carolina. To mitigate STI transmission in the context of HIV, targeted interventions for high-risk populations are needed.

**Supplementary Information:**

The online version contains supplementary material available at 10.1007/s10461-025-04744-5.

## Introduction

The U.S. Centers for Disease Control and Prevention (CDC) reported that nearly half of new HIV infections in 2022 occurred in the Southern United States (US) [1]. The Ending the HIV Epidemic (EHE) initiative aims to reduce new HIV infections by at least 90% by 2030. Due to its high rural HIV burden, South Carolina (SC) is designated as a priority jurisdiction area under the EHE initiative, with rurality posing a significant challenge to achieving this goal. In 2021, SC ranked ninth amongst US states for HIV incidence, with 15 new cases per 100,000 population [2].

Sexually transmitted infections (STIs) are a known risk factor for HIV acquisition and create significant challenges to achieving the goals of the EHE initiative [3]. The relationship between STIs and HIV is bidirectional, as the presence of one infection influences the other. HIV-associated ulcerative lesions and a compromised immune system from HIV infection can increase susceptibility to STIs among people with HIV (PWH) [4, 5]. Conversely, STIs can cause local inflammation in the genital tract, facilitating HIV shedding and increasing the risk of HIV transmission [6]. A systematic review of studies conducted across various geographical settings reported an average point prevalence of STIs among PWH of 16.3% [5]. Additionally, a study of the entire population of PWH in New York City found that 2.4% were diagnosed with new cases of gonorrhea, chlamydia, or syphilis within one year [7]. The STI infection rate in PWH is significantly higher than that of the general population [8, 9].

Various factors contribute to the risk of STIs among PWH. Younger individuals are at greater risk, possibly due to having more sexual partners and inconsistent use of protective measures [4, 7, 10–15]. African-American and Hispanic individuals are at increased risk due to limited access to health care and living in areas with high STI prevalence [7, 10, 11]. Men who have sex with men (MSM) and transgender women also face a higher risk, although this may partly be due to more frequent testing within these groups [7, 11–13, 15]. Drug use, whether injecting or non-injecting, is linked to increased STI risk due to associated risk behaviors such as condomless sex [4, 13, 16, 17]. A history of casual sexual relationships and prior STI infection further elevates STI risk [4, 15]. Additionally, higher CD4 + cell counts are associated with riskier sexual behaviors, while not receiving antiretroviral therapy (ART) is linked to an increased risk of STIs, further highlighting the bidirectional relationship between HIV and STIs [11, 13, 14].

Most studies examining STIs among PWH have primarily targeted specific groups at high risk for HIV transmission, like MSM, resulting in limited data on the long-term incidence of STIs in broad, representative cohorts. Additionally, the increasing rates of STI infections documented across the US, including among PWH, highlight the need for comprehensive studies on the risk factors associated with incident STI infections among PWH [12, 15, 18]. This study will leverage unique linked South Carolina HIV/AIDS and STI data to examine temporal trends in STI incidence from 2007 to 2020 and assess the associated risk factors within a statewide representative cohort of PWH. We hypothesize an increase in STI incidence during the study period and expect to find disparities in STI risk influenced by various demographic and clinical factors, like age, race/ethnicity, and HIV transmission risk group.

## Methods

### Study Population and Data Sources

This study included all adults (≥ 18 years old) diagnosed with HIV in South Carolina from January 2007 to April 2018 (*N* = 8,015). The de-identified data, encompassing HIV diagnosis dates, demographic characteristics, modes of HIV transmission, and clinical information, were extracted from the South Carolina HIV/AIDS electronic reporting system (e-HARS) in the Department of Public Health. Although minors are represented in the surveillance system, the dataset available for analysis comprised only adults. This surveillance database has recorded statewide CD4+ cell count and viral load (VL) test results since 2004.

The data was linked with the Sexually Transmitted Diseases Management Information System (STDMIS), which collects data on all notifiable sexually transmitted diseases in South Carolina, including syphilis, chlamydia, and gonorrhea. The study included STI diagnoses between January 2005 and April 2021, covering two years before HIV diagnosis and at least three years of follow-up post-diagnosis. The South Carolina Department of Public Health (SC DPH) linked and de-identified these databases.

### Outcome

The primary outcome of interest was the incidence of the first STI (chlamydia, gonorrhea, or syphilis) following an HIV diagnosis. All participants diagnosed with at least one of these STIs after their HIV diagnosis were classified as having an incident STI. For individuals with multiple STI diagnoses, only the first was used in the analysis. Each PWH was followed from the date of HIV diagnosis until the first occurrence of an STI diagnosis, death, or the end of the study period (April 2021), whichever occurred first. Annual incidence rates were presented as the incidence rate per 100 person-years, calculated by dividing the number of new cases by the total follow-up time among the population at risk and multiplying by 100.

Secondary outcomes of interest included the time to first STI diagnosis and the risk factors for STI infection, stratified by the type of STI.

### Covariates

The demographic characteristics considered were age at HIV diagnosis (18–29, 30–39, 40–49, and ≥ 50 years), gender (male, female), and race/ethnicity (Black, White, Hispanic, other/unknown). HIV transmission modes included heterosexual, MSM, injection drug users (IDU), and other/unknown. Residence at HIV diagnosis was categorized into rural and urban areas according to the 2013 USDA Rural-Urban Continuum Codes, with codes 1–3 representing urban areas and 4–9 representing rural areas.

Clinical characteristics included initial VL at diagnosis (< 200; 200 − 10,000; 10,000–100,000; >100,000 copies/ml), and initial CD4 + cell count at diagnosis (< 200; 200–350; >350 copies/mm3). PWH without VL or CD4 + measurements within 90 days of HIV diagnosis were categorized as unknown.

### Statistical Analysis

We used descriptive statistics to examine demographic and clinical cohort characteristics. Between and within-group comparisons for groups with and without STIs were done using chi-squared tests. We applied a Cox Proportional Hazards (PH) model to estimate the relationship between the time to first STI diagnosis following HIV diagnosis and various demographic and clinical factors. The PH assumption was validated by plotting the logarithm of the cumulative hazard function against the follow-up time. We conducted a sensitivity analysis that excluded adults diagnosed with an STI in the year prior to their HIV diagnosis to ensure that the STI diagnosis was new and not due to persistent or recurrent infections. All analyses were performed using SAS version 9.4 (SAS Institute Inc., Cary, NC) and R version 4.1.2 (R Foundation for Statistical Computing, Vienna, Austria) statistical software.

## Results

### Sample Characteristics

The sample included 8,015 participants diagnosed with HIV, of whom 28.73% contracted at least one STI during the follow-up period. The mean follow-up time was 2,339.1 days for the entire cohort and 1,105.5 days for individuals who contracted an STI after HIV diagnosis. The study found significant differences between individuals with and without an STI diagnosis based on various demographic and clinical characteristics (Table 1). Notably, the age distribution varied significantly; individuals aged 18–29 years comprised 66.91% of those diagnosed with an STI, whereas only 31.88% fell into this age range in the non-STI group (*p* < 0.0001). Males were significantly overrepresented among those with an STI diagnosis, making up 87.76% versus 72.13% in the non-STI cohort (*p* < 0.0001). Black PWH were disproportionately affected, comprising 77.38% of those with an initial STI diagnosis compared to 66.95% of those without an STI diagnosis (*p* < 0.0001). The mode of HIV transmission indicated that MSM accounted for 75.38% of initial STI cases diagnosed after HIV, a significantly larger proportion (*p* < 0.0001) compared to 43.22% in the group without STIs. Urban residence was slightly more common among those diagnosed with an STI (84.98%) compared to those not diagnosed with an STI (82.91%) (*p* = 0.0243).

PWH diagnosed with an STI had lower initial VL (Table 1), with 27.66% having a VL between 200–10,000 copies/ml, compared to 24.30% in the non-STI group (*p* < 0.0001). Higher initial CD4+ cell counts (> 350 cells/mm³) were more common in the STI group (47.94%) than in the non-STI group (39.79%) (*p* < 0.0001). 

**Table 1:** Demographic characteristics of South Carolina PWH by STI diagnosis status

Characteristic		Overall (*N* = 8015)	No STI (*N* = 5712)	Any STI (*N* = 2303)	*P*-value
Age	18–29	3,362 (41.95)	1,821 (31.88)	1,541 (66.91)	< 0.0001
	30–39	1,662 (20.74)	1,244 (21.78)	418 (18.15)	
	40–49	1,614 (20.14)	1,365 (23.90)	249 (10.81)	
	50+	1,377 (17.18)	1,282 (22.44)	95 (4.13)	
Gender	Female	1,874 (23.38)	1,592 (27.87)	282 (12.24)	< 0.0001
	Male	6,141 (76.62)	4,120 (72.13)	2,021 (87.76)	
Race	White	1,736 (21.66)	1,343 (23.51)	393 (17.06)	< 0.0001
	Black	5,606 (69.94)	3,824 (66.95)	1,782 (77.38)	
	Hispanic	455 (5.68)	374 (6.55)	81 (3.52)	
	Other/unknown	218 (2.72)	171 (2.99)	47 (2.04)	
Transmission risk	Heterosexual	1,485 (18.53)	1,249 (21.87)	236 (10.25)	< 0.0001
	MSM	4,205 (52.46)	2,469 (43.22)	1,736 (75.38)	
	IDU/MSM	415 (5.18)	320 (5.60)	95 (4.13)	
	Other/unknown	1,910 (23.83)	1,674 (29.31)	236 (10.25)	
Residence at HIV diagnosis	Rural	1,322 (16.49)	976 (17.09)	346 (15.02)	0.0243
	Urban	6,693 (83.51)	4,736 (82.91)	1,957 (84.98)	
Initial VL count	< 200	231 (2.88)	204 (3.57)	27 (1.17)	< 0.0001
	200 − 10,000	2,025 (25.27)	1,388 (24.30)	637 (27.66)	
	10,000–100,000	2,655 (33.13)	1,815 (31.78)	840 (36.47)	
	> 100,000	2,118 (26.43)	1,591 (27.85)	527 (22.88)	
	Unknown	986 (12.30)	714 (12.50)	272 (11.81)	
Initial CD4 count	< 200	2,163 (26.99)	1,773 (31.04)	390 (16.93)	< 0.0001
	200–350	1,483 (18.50)	973 (17.03)	510 (22.15)	
	> 350	3,377 (42.13)	2,273 (39.79)	1,104 (47.94)	
	Unknown	992 (12.38)	693 (12.13)	299 (12.98)	

### STI Incidence Temporal Trends

**Fig. 1:**
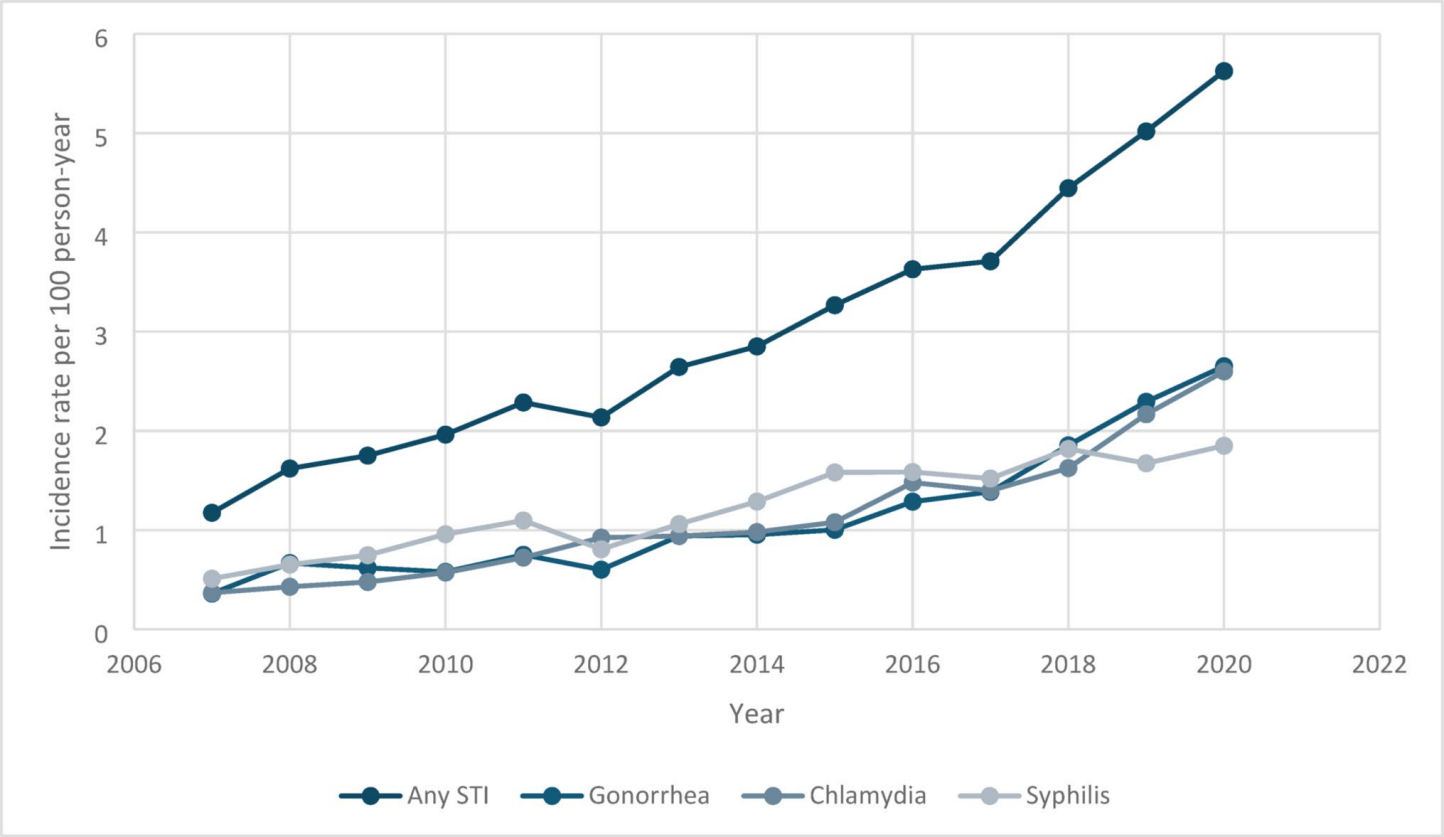
Trends in Gonorrhea, Chlamydia, and Syphilis incidence for PWH in South Carolina (2007–2020)

The incidence rate of initial STI diagnoses following an HIV diagnosis displayed a consistent upward trend throughout the study period, increasing from approximately 1.2 per 100 person-years in 2007 to over 5.5 per 100 person-years by 2020 (Fig. 1). Notably, the diagnoses of chlamydia and gonorrhea as initial STIs after HIV diagnosis showed significant increases over time. The incidence rate of chlamydia began at 0.37 per 100 person-years in 2007 and steadily rose to nearly 2.6 per 100 person-years by 2020. Similarly, gonorrhea’s incidence rate climbed from 0.36 per 100 person-years in 2007 to about 2.7 per 100 person-years by 2020. Syphilis also exhibited an upward trend, albeit with more variability. It started at 0.5 per 100 person-years and increased to 1.8 per 100 person-years by 2018. Following a slight decrease to 1.7 in 2019, the incidence rate of initial syphilis diagnoses rose again in 2020. Overall, these findings indicate a significant and sustained increase in initial STI diagnoses over the 13-year period.

### Time to Initial STI Diagnosis

**Fig. 2:**
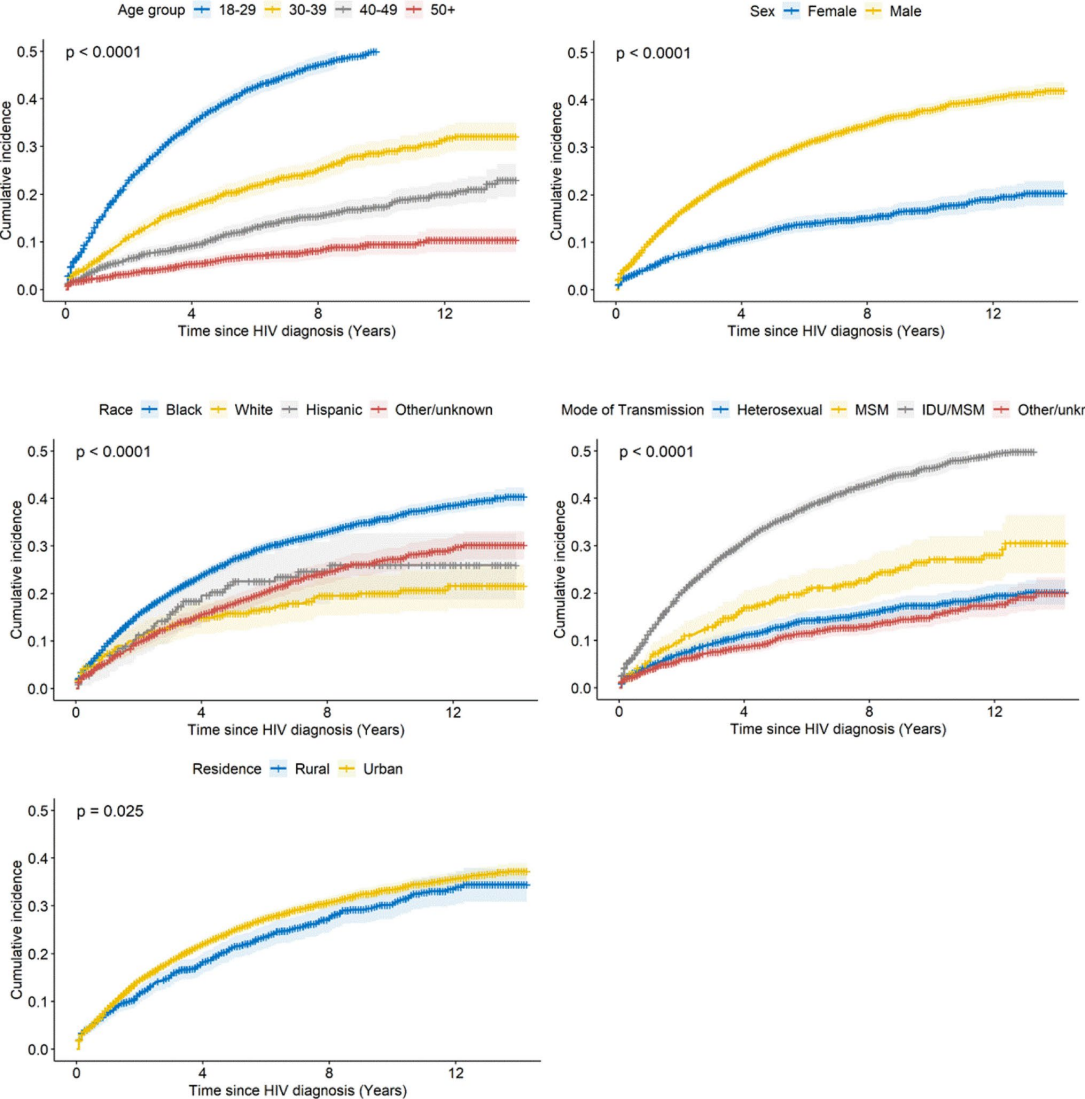
Cumulative incidence of chlamydia, gonorrhea or syphilis by demographic characteristics for PWH in South Carolina

The overall cumulative incidence of a first STI (gonorrhea, chlamydia, or syphilis) after HIV diagnosis varied significantly by demographic and behavioral factors (Fig. 2). Age group analysis showed a decreasing risk of an STI diagnosis with increasing age. About 52.6% (95% CI: 50.5–54.6) of those aged 18–29 had an initial STI diagnosis within the 12-year follow-up period, compared to 10.4% (95% CI: 8.0–12.7) among those aged 50 and older (*P* < 0.0001). Younger individuals (aged 18–29) also experienced a shorter time to an initial STI diagnosis, with a median time to diagnosis of 760 days, versus 1,095 days for those aged 40–49. Men had a higher risk of an initial STI diagnosis than women, with 40.4% (95% CI: 36.9–40.1) of men versus 19.0% (95% CI: 16.7–21.2) of women diagnosed with an STI (*P* < 0.0001). Men also had a shorter time to initial STI diagnosis, with a median time to diagnosis of 795 days compared to 825 days for women. Significant racial disparities were observed, with Black individuals having a cumulative incidence of 38.6% (95% CI: 37.0–40.1), compared to 21.6% (95% CI: 16.9–26.0) for White individuals (*P* < 0.0001). Interestingly, Hispanic individuals experienced a high risk of STI diagnosis within the first 5–6 years post-HIV diagnosis, but this risk leveled off. Racial differences persisted throughout the time to first STI diagnosis. White individuals had a median follow-up time of 945 days before developing an STI after HIV diagnosis, whereas Black (790 days) and Hispanic individuals (580 days) had shorter times to first STI.

Individuals with a history of IDU showed the highest cumulative incidence (Fig. 2), at approximately 49.3% (95% CI: 47.4–51.2) after 12 years of follow-up, while MSM had a cumulative incidence of 28.0% (95% CI: 22.6–33.1) (*P* < 0.0001). Urban residents had a slightly higher cumulative incidence of initial STI diagnoses (*P* = 0.025) and a shorter median time to first STI diagnosis than rural residents (790 days vs. 885 days). 

### Risk Factors

The analysis identified multiple demographic and clinical factors associated with an increased risk of an STI diagnosis, including gonorrhea, chlamydia, and syphilis (Table 2). Younger age was a significant predictor, with those aged 18–29 having the highest risk of an initial STI diagnosis compared to individuals over 50 (aHR = 4.496 for any STI, aHR = 6.432 for gonorrhea, aHR = 6.229 for chlamydia, aHR = 2.795 for syphilis; all p-values < 0.0001). Risk declined with increasing age but remained elevated for those aged 30–39 (aHR = 2.771 for any STI) and 40–49 (aHR = 1.797 for any STI) relative to the oldest group. Men had a lower likelihood of an initial chlamydia infection (aHR = 0.725, *p* = 0.0068) but were at significantly higher risk for an initial syphilis infection (aHR = 4.190, *p* < 0.0001) compared to women. Black individuals had increased risks for an initial STI diagnosis across all STIs (aHR = 1.407 for any STI, *p* < 0.0001), while Hispanic individuals and those in the “Other/Unknown” race category did not show significant differences from White individuals. MSM had significantly higher risks for an initial diagnosis of all STIs, particularly gonorrhea (aHR = 2.048, *p* < 0.0001) and syphilis (aHR = 5.361, *p* < 0.0001), compared to heterosexual individuals. IDUs also had increased risks for gonorrhea (aHR = 1.600, *p* = 0.0158) and syphilis (aHR = 3.494, *p* < 0.0001). Urban residence was associated with a slightly higher risk of any STI (aHR = 1.127, *p* = 0.0408) but was not statistically significant for specific STIs.

Regarding clinical factors, higher initial VL was associated with increased initial STI diagnosis risk (Table 2), with the highest risk observed for those with VL > 100,000 copies/mL (aHR = 1.913, *p* = 0.0012 for any STI). Generally, a higher initial VL was associated with a higher STI diagnosis risk. Initial CD4+ cell count was also linked to STI risk, with individuals having CD4+ cell counts between 200 and 350 cells/µL (aHR = 1.472, *p* < 0.0001) and > 350 cells/µL (aHR = 1.465, *p* < 0.0001) at higher risk compared to those with CD4+ < 200 cells/µL for any STI. 

**Table 2:** Risk factors associated with incident stis among PWH in South Carolina

Characteristics		Any STI		Gonorrhea		Chlamydia		Syphilis	
		aHR (95% CI)	P-value	aHR (95% CI)	P-value	aHR (95% CI)	P-value	aHR (95% CI)	P-value
Age	50+	Ref.		Ref.		Ref.		Ref.	
	18–29	4.496 (3.628,5.571)	< 0.0001	6.432 (4.476,9.244)	< 0.0001	6.229 (4.475,8.670)	< 0.0001	2.795 (2.086,3.745)	< 0.0001
	30–39	2.771 (2.213,3.471)	< 0.0001	3.115 (2.128,4.559)	< 0.0001	2.872 (2.024,4.075)	< 0.0001	2.264 (1.667,3.075)	< 0.0001
	40–49	1.797 (1.418,2.278)	< 0.0001	1.475 (0.971,2.241)	0.0685	1.744 (1.201,2.532)	0.0035	1.687 (1.225,2.322)	0.0013
Gender	Female	Ref.		Ref.		Ref.		Ref.	
	Male	1.083 (0.909,1.290)	0.3721	1.043 (0.796,1.365)	0.7618	0.725 (0.575,0.915)	0.0068	4.190 (2.723,6.448)	< 0.0001
Race	White	Ref.		Ref.		Ref.		Ref.	
	Black	1.407 (1.256,1.577)	< 0.0001	1.714 (1.433,2.051)	< 0.0001	1.500 (1.264,1.778)	< 0.0001	1.446 (1.241,1.683)	< 0.0001
	Hispanic	0.815 (0.640,1.038)	0.0967	0.829 (0.564,1.218)	0.3386	1.055 (0.756,1.472)	0.7532	0.751 (0.533,1.057)	0.1009
	Other/unknown	1.015 (0.749,1.375)	0.9249	1.202 (0.767,1.883)	0.4216	1.145 (0.746,1.759)	0.5359	1.396 (0.967,2.016)	0.0753
Transmission risk	Heterosexual	Ref.		Ref.		Ref.		Ref.	
	IDU	1.593 (1.236,2.053)	0.0003	1.600 (1.092,2.344)	0.0158	1.033 (0.706,1.511)	0.8664	3.494 (2.232,5.472)	< 0.0001
	MSM	2.181 (1.814,2.622)	< 0.0001	2.048 (1.551,2.704)	< 0.0001	1.613 (1.254,2.074)	0.0002	5.361 (3.710,7.749)	< 0.0001
	Other/unknown	0.908 (0.754,1.094)	0.3085	0.800 (0.602,1.064)	0.1252	0.961 (0.761,1.214)	0.7407	1.286 (0.850,1.946)	0.2331
Residence at HIV diagnosis	Rural	Ref.		Ref.		Ref.		Ref.	
	Urban	1.127 (1.005,1.265)	0.0408	1.078 (0.917,1.267)	0.3606	1.154 (0.980,1.359)	0.0857	1.127 (0.964,1.317)	0.1347
Initial VL	< 200	Ref.		Ref.		Ref.		Ref.	
	200 − 10,000	1.572 (1.068,2.314)	0.0218	1.372 (0.787,2.392)	0.2652	1.140 (0.698,1.861)	0.6005	1.700 (0.975,2.962)	0.0612
	10,000–100,000	1.733 (1.178,2.549)	0.0052	1.450 (0.832,2.526)	0.1899	1.407 (0.863,2.293)	0.1706	1.657 (0.952,2.885)	0.0742
	> 100,000	1.913 (1.292,2.830)	0.0012	1.583 (0.900,2.783)	0.111	1.599 (0.972,2.632)	0.0647	1.866 (1.064,3.272)	0.0294
	Unknown	1.372 (0.897,2.099)	0.1449	1.214 (0.657,2.245)	0.5364	1.045 (0.603,1.810)	0.8757	1.342 (0.734,2.454)	0.3387
Initial CD4	< 200	Ref.		Ref.		Ref.		Ref.	
	200–350	1.472 (1.283,1.688)	< 0.0001	1.501 (1.231,1.831)	< 0.0001	1.950 (1.592,2.389)	< 0.0001	1.234 (1.028,1.482)	0.0242
	> 350	1.465 (1.290,1.664)	< 0.0001	1.374 (1.139,1.658)	0.0009	1.818 (1.499,2.204)	< 0.0001	1.273 (1.075,1.507)	0.0051
	Unknown	1.544 (1.248,1.911)	< 0.0001	1.342 (0.978,1.841)	0.0681	1.862 (1.371,2.530)	< 0.0001	1.526 (1.153,2.020)	0.0031

Overall, the trends remain consistent in the sensitivity analysis, with younger age groups, MSM, and individuals with higher initial VL continuing to have an increased risk of STIs (Appendix A).

## Discussion

This study provides vital insights into the incidence of initial STI diagnoses among PWH in South Carolina, revealing important trends and disparities that could impact public health strategies. The findings reveal a notable increase in the incidence of initial STI diagnoses among PWH from 2007 to 2020, reflecting national trends and underscoring the urgent need to incorporate STI prevention as part of the EHE initiative.

A plausible explanation for the rise in STI rates after an HIV diagnosis is the introduction of pre-exposure prophylaxis (PrEP) as recommended by the CDC and US Preventive Services Task Force [19–21]. Research has shown a global link between PrEP and higher STI rates, likely due to risk compensation, where individuals engage in riskier behaviors because they feel less at risk [22, 23]. This phenomenon is especially notable among MSM, with studies indicating that PWH who have a partner on PrEP experience nearly three times the prevalence of STIs compared to those with HIV-negative partners not taking PrEP [24]. However, other studies show that PrEP facilitates early STI screening among users, benefiting their partners, including PWH, by reducing transmission [25, 26].

The results show that younger PWH, particularly those aged 18–29, are most at risk for STIs, consistent with previous studies showing they engage in riskier sexual behaviors and have more partners [4, 7, 10–15]. Contributing factors include inconsistent condom use, substance use, and poor sex education [27–30]. Additionally, the data reveals that Black PWH experience a significantly higher risk for STIs than White PWH, plausibly influenced by structural racism, poverty, mistrust in healthcare, and risky behaviors [31, 32]. Other concerns about healthcare quality and confidentiality also contribute to higher STI rates among Black PWH [31–33].

MSM had higher risks for STIs, particularly gonorrhea and syphilis, which is consistent with prior studies identifying MSM as a pivotal group in the STI epidemic among PWH [7, 11–13, 15]. While increased testing within this population may partly explain the higher rate of STI infections, other factors such as decreased condom usage, increased risk-taking due to HIV treatment, and reduced access to primary care have also been reported [34]. The association between urban residency and increased STI risk suggests that environmental factors like greater population density and larger sexual networks may contribute to this trend. Inconsistent with prior research in South Carolina is the finding that urban PWHs are at higher risk for STIs that rural PWH [33]. Further research is needed to understand these findings and their implications better.

The clinical profile of PWH in this study indicates that higher initial CD4+ cell counts and VLs are associated with increased STI risk, highlighting the complex relationship between HIV and STIs. Higher initial VL may increase susceptibility to STI due to compromised immune systems and increased viral shedding [4, 5]. Although prior research has associated higher initial CD4+ cell counts with increased high-risk sexual behaviors due to improved well-being, further investigation is warranted to elucidate this link [11].

The observed trends emphasize the need to incorporate STI prevention and treatment into HIV programs, especially for high-risk groups such as young people, racial minorities, and MSM. Although programs that integrate STI and HIV services exist, significant gaps remain [35]. Comprehensive sexual health education and ongoing promotion of safe sex are crucial in reducing STI risk among PWH. Current STI syndromic surveillance might be inadequate for screening and early detection. Enhanced healthcare access and tailored interventions targeting racial disparities have been shown to reduce the incidence of STIs in affected populations [36, 37].

Although significant, this study acknowledges its limitations. First, its retrospective nature may introduce data completeness and accuracy biases. Reliance on public health databases limits clinical details like STI screening frequency or behavioral risk factors, which could provide further insights into the observed trends. The study focuses on South Carolina, potentially not representing PWH across the US, limiting generalizability, but more representative of Southern states. Lastly, despite controlling for various factors, unmeasured confounders such as mental health, socioeconomic status, and substance use could impact both STI acquisition and health-seeking behaviors.

In conclusion, this study underscores the rising rate of STIs among PWH in South Carolina and the related demographic and clinical risk factors. Further integrating STI management within HIV care is vital to meeting the objectives of the Ending the HIV Epidemic initiative. More investigation is needed to examine long-term trends in STI rates and evaluate the effectiveness of specific interventions for high-risk groups. Gaining insights into these patterns is essential for developing holistic public health strategies to decrease both HIV and STI transmission in susceptible populations.

## Electronic Supplementary Material

Below is the link to the electronic supplementary material.

Supplementary Material 1

## Data Availability

Data not publicly available.
