# Supplementary material for: Sexually Transmitted Infection (STI) Incidence and Risk Factors Among People with HIV (PWH): Insights from a 13-Year Cohort Study in South Carolina
Source: AIDS Behav. 2025 Apr 24;29(9):2882–90. doi: 10.1007/s10461-025-04744-5 (PMC12431878; doi:10.1007/s10461-025-04744-5)
Supplement: Supplementary file 1 — Supplementary Material 1 [file 10461_2025_4744_MOESM1_ESM.docx]

Appendix A.

| Characteristics | | Any STI | | Gonorrhea | | Chlamydia | | Syphilis | |
| --- | --- | --- | --- | --- | --- | --- | --- | --- | --- |
|  |  | **aHR**  **(95% CI)** | **P-value** | **aHR**  **(95% CI)** | **P-value** | **aHR**  **(95% CI)** | **P-value** | **aHR**  **(95% CI)** | **P-value** |
| Age | 50+ | Ref. |  | Ref. |  | Ref. |  | Ref. |  |
|  | 18-29 | 4.496 (3.628,5.571) | <0.0001 | 6.432 (4.476,9.244) | <0.0001 | 6.229 (4.475,8.670) | <0.0001 | 2.795 (2.086,3.745) | <0.0001 |
|  | 30-39 | 2.771 (2.213,3.471) | <0.0001 | 3.115 (2.128,4.559) | <0.0001 | 2.872 (2.024,4.075) | <0.0001 | 2.264 (1.667,3.075) | <0.0001 |
|  | 40-49 | 1.797 (1.418,2.278) | <0.0001 | 1.475 (0.971,2.241) | 0.0685 | 1.744 (1.201,2.532) | 0.0035 | 1.687 (1.225,2.322) | 0.0013 |
| Gender | Female | Ref. |  | Ref. |  | Ref. |  | Ref. |  |
|  | Male | 1.083 (0.909,1.290) | 0.3721 | 1.043 (0.796,1.365) | 0.7618 | 0.725 (0.575,0.915) | 0.0068 | 4.190 (2.723,6.448) | <0.0001 |
| Race | White | Ref. |  | Ref. |  | Ref. |  | Ref. |  |
|  | Black | 1.407 (1.256,1.577) | <0.0001 | 1.714 (1.433,2.051) | <0.0001 | 1.500 (1.264,1.778) | <0.0001 | 1.446 (1.241,1.683) | <0.0001 |
|  | Hispanic | 0.815 (0.640,1.038) | 0.0967 | 0.829 (0.564,1.218) | 0.3386 | 1.055 (0.756,1.472) | 0.7532 | 0.751 (0.533,1.057) | 0.1009 |
|  | Other/unknown | 1.015 (0.749,1.375) | 0.9249 | 1.202 (0.767,1.883) | 0.4216 | 1.145 (0.746,1.759) | 0.5359 | 1.396 (0.967,2.016) | 0.0753 |
| Transmission risk | Heterosexual | Ref. |  | Ref. |  | Ref. |  | Ref. |  |
|  | IDU | 1.593 (1.236,2.053) | 0.0003 | 1.600 (1.092,2.344) | 0.0158 | 1.033 (0.706,1.511) | 0.8664 | 3.494 (2.232,5.472) | <0.0001 |
|  | MSM | 2.181 (1.814,2.622) | <0.0001 | 2.048 (1.551,2.704) | <0.0001 | 1.613 (1.254,2.074) | 0.0002 | 5.361 (3.710,7.749) | <0.0001 |
|  | Other/unknown | 0.908 (0.754,1.094) | 0.3085 | 0.800 (0.602,1.064) | 0.1252 | 0.961 (0.761,1.214) | 0.7407 | 1.286 (0.850,1.946) | 0.2331 |
| Residence at HIV diagnosis | Rural | Ref. |  | Ref. |  | Ref. |  | Ref. |  |
|  | Urban | 1.127 (1.005,1.265) | 0.0408 | 1.078 (0.917,1.267) | 0.3606 | 1.154 (0.980,1.359) | 0.0857 | 1.127 (0.964,1.317) | 0.1347 |
| Initial VL | <200 | Ref. |  | Ref. |  | Ref. |  | Ref. |  |
|  | 200-10,000 | 1.572 (1.068,2.314) | 0.0218 | 1.372 (0.787,2.392) | 0.2652 | 1.140 (0.698,1.861) | 0.6005 | 1.700 (0.975,2.962) | 0.0612 |
|  | 10,000-100,000 | 1.733 (1.178,2.549) | 0.0052 | 1.450 (0.832,2.526) | 0.1899 | 1.407 (0.863,2.293) | 0.1706 | 1.657 (0.952,2.885) | 0.0742 |
|  | >100,000 | 1.913 (1.292,2.830) | 0.0012 | 1.583 (0.900,2.783) | 0.111 | 1.599 (0.972,2.632) | 0.0647 | 1.866 (1.064,3.272) | 0.0294 |
|  | Unknown | 1.372 (0.897,2.099) | 0.1449 | 1.214 (0.657,2.245) | 0.5364 | 1.045 (0.603,1.810) | 0.8757 | 1.342 (0.734,2.454) | 0.3387 |
| Initial CD4 | <200 | Ref. |  | Ref. |  | Ref. |  | Ref. |  |
|  | 200-350 | 1.472 (1.283,1.688) | <0.0001 | 1.501 (1.231,1.831) | <0.0001 | 1.950 (1.592,2.389) | <0.0001 | 1.234 (1.028,1.482) | 0.0242 |
|  | >350 | 1.465 (1.290,1.664) | <0.0001 | 1.374 (1.139,1.658) | 0.0009 | 1.818 (1.499,2.204) | <0.0001 | 1.273 (1.075,1.507) | 0.0051 |
|  | Unknown | 1.544 (1.248,1.911) | <0.0001 | 1.342 (0.978,1.841) | 0.0681 | 1.862 (1.371,2.530) | <0.0001 | 1.526 (1.153,2.020) | 0.0031 |
